# Painting from Life Nature's Unpredictable Menagerie

**DOI:** 10.3201/eid1112.AC1112

**Published:** 2005-12

**Authors:** Polyxeni Potter

**Affiliations:** * Centers for Disease Control and Prevention, Atlanta, Georgia, USA

**Keywords:** Art and science, emerging infectious diseases,Bruegel, Brueghel, Entry of Animals into Noah's Ark, zoonotic disease, multisector alliances

**Figure:**
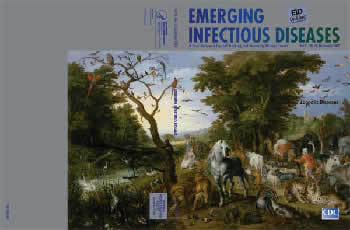
Jan Brueghel the Elder (1568–1625). The Entry of the Animals into Noah's Ark (1613). Oil on panel (54.6 cm × 83.8 cm). The J. Paul Getty Museum, Los Angeles, California, USA (92.P8.82). Courtesy of the J. Paul Getty Museum

"On his journeys Bruegel did many views from nature, so it was said of him when he traveled through the Alps that he had swallowed all the mountains and rocks and spat them out again, after his return, onto his canvases and panels, so closely was he able to follow nature here and in his other works" (*1*). This brilliant legacy, become familial burden, framed the life and work of Jan Brueghel the Elder, Pieter Bruegel's^1^ son, and his sons after him. Always measured against the original, "Peasant" Bruegel, descendants in this legendary family held their own, each making a mark, all painstakingly distinguishing themselves through the choice of subject matter and niceties of style.

Jan Brueghel hardly knew his father. Orphaned soon after his birth in Brussels, he studied with Pieter Goctkind and Gillis van Coninxloo in Antwerp, learned watercolor painting from his grandmother Mayken Verhulst, and flourished under the patronage of great collector Cardinal Federigo Borromeo in Rome and Milan. Although he grew up copying his father's works, he was influenced little by them or those of his brother, Pieter Brueghel the Younger, called "Hell" Brueghel for his fiery depictions of afterlife (*2*).

Art in the Low Countries during the 1600s was dominated by the Brueghel family, who worked in Antwerp amidst political and social change. The spread of humanism affected popular tastes, favoring mythological over religious themes in the visual arts. And with commissions by the church, court, and nobility on the decline, painting specialties (genre, still life, landscape) appealing to patrons of more modest means became popular. The Brueghels so excelled in the new specialties that they created a trend for their generation, a bridge between the technical refinement of Flemish primitive art and the expansive imagination seen later in the work of Peter Paul Rubens and his followers (*3*).

Jan became known as "Flower" Brueghel, even though he started painting flowers late in his career. Tulips, hyacinths, marigolds, nasturtiums, and sunflowers were as new in Europe as the artistic genre they embellished. With a modern insistence on painting from nature, the artist traveled far to find flora for his lush scenes. Botanical specimens of various seasons often appeared together in bucolic Eden-like scenes that earned him another name, "Paradise" Brueghel. As was the custom, figures in his scenes were sometimes painted by other artists. Rubens, a close friend, was a frequent collaborator, as with Madonna in a Wreath of Flowers for which Brueghel painted the iconic wreath. Jan Brueghel II (1601–1678) and Ambrosius Brueghel (1617–1675) continued the tradition of flower still life long after their father's death of cholera in Antwerp.

Jan Brueghel painted on various media, among them copper, an innovation learned during his tenure in Italy and exploited to full advantage in hundreds of paintings. The smoothness of copper allowed the brush to glide on the surface without the interruption or absorption characteristic of wood or canvas surfaces. Close-up forms were painted with visible brushstrokes of thick paint, distant ones with fluid, thinly diluted paint. Even the minutest figures in the artist's tightly structured compositions were distinguishable (*4*). Meticulous attention to detail and ability to control the brush and create surfaces of exquisite refinement and sheen earned Jan his most common name, "Velvet" Brueghel.

The Entry of Animals into Noah's Ark, on this month's cover, was methodically assembled. The sprawling backdrop was filled with detailed vegetation, for which the artist had become famous and which secured his legacy during his lifetime. The scene teemed with nature's creatures, domestic and wild, from the tiniest to the most imposing, painted from life at Infanta Isabella's menagerie of exotic animals in Brussels (*5*). Reminiscent of other Jan Brueghel paintings of animals in nature, the tableau reflected the interest and curiosity about natural history sparked by discovery of the New World and its exotic plant and animal life.

Affection and concern for animals were also central to ark lore and its countless interpretations. When biblical balance and harmony broke down and precipitated the flood, animals were invited to the ark, as if world survival would have been unthinkable without them. Assembled in this unreal scene in their most realistic attire, they seemed unaware of the importance of the occasion. Oblivious to the clouds building in the horizon, many strayed from the shepherded line moving toward the ark in the far distance. Distracted, churlish, and unruly, they seized a moment of human inattention to wander off into mayhem.

Jan Brueghel's rendition of biblical survival seems allegorical of emerging zoonoses. As in this animal-human gathering, in nature, balance and harmony are imperiled by irregularity or unpredictable biological behavior for which no host defenses are immediately available. And like shepherding skills, existing protective mechanisms can be overwhelmed by unexpected turns. Biological and social systems and infrastructures prove inadequate against new agents and modes of transmission and demand new measures and approaches; among them, multisector alliances able to bridge the gap in public health response between recognition and control of new hazards to humans and animals (*6**,**7*). Above all, closely following nature, proven to make better art, also makes better defense against emerging diseases.
